# Combined Posterior Tibial Tendon Transfer and Forefoot Reconstruction for Foot Drop and Forefoot Pain Secondary to Charcot-Marie-Tooth Disease: A Case Report and Literature Review

**DOI:** 10.7759/cureus.100753

**Published:** 2026-01-04

**Authors:** Mark H Moss, Angela M Garcia

**Affiliations:** 1 Podiatry, Coastal Foot and Ankle Associates, Pearland, USA; 2 Podiatry, HCA Houston Healthcare West Residency Program, Houston, USA

**Keywords:** charcot-marie-tooth disease, drop foot, interosseous membrane, outcome measure, posterior tibial tendon

## Abstract

Foot drop is a debilitating condition characterized by impaired ankle dorsiflexion and is commonly associated with neuromuscular disorders such as Charcot-Marie-Tooth (CMT) disease. CMT1A, the most prevalent subtype, leads to progressive weakness of the anterior compartment musculature and a cavovarus deformity. Posterior tibial tendon (PTT) transfer remains a well-established surgical technique for restoring active dorsiflexion in cases of irreversible foot drop. We present the case of a 59-year-old female with CMT1A, diabetes mellitus, and peripheral neuropathy who developed progressive left-sided foot drop. Conservative treatment, including a custom ankle-foot orthosis (AFO), was initially attempted. Following failed conservative management, the patient underwent a PTT transfer through the interosseous membrane with interference screw fixation into the intermediate cuneiform. This was combined with a tendoachilles lengthening, hammertoe correction, and Weil osteotomies of the second and third metatarsals. Clinically, she achieved 4° of dorsiflexion above neutral, transitioned to regular footwear, discontinued AFO use, and returned to full functional activity. This case report reinforces the role of PTT transfer as an effective, single-stage reconstructive option for neuromuscular foot drop when combined with appropriate adjunctive procedures and structured rehabilitation.

## Introduction

Foot drop is a debilitating deformity characterized by impaired dorsiflexion at the tibiotalar joint. It most commonly results from neuromuscular disease or injury to the common peroneal nerve. The resulting gait disturbance significantly impairs functional mobility and quality of life. Charcot-Marie-Tooth (CMT) disease is a progressive hereditary peripheral neuropathy caused by defective myelination of peripheral nerves. The most common form is CMT type 1, with subtype 1A linked to a duplication of the PMP22 gene on chromosome 17 [1]. Patients with CMT1A typically present with calcaneal varus foot deformity and weakness of the tibialis anterior and peroneal muscle groups.

Management of CMT-associated foot deformities depends on the functional status of the remaining musculature and the progressive nature of the disease. Conservative options include orthotics, custom ankle-foot orthoses (AFOs), and physical therapy. Surgical intervention may be indicated based on deformity flexibility, patient goals, and neuromuscular function. Multiple tendon transfer procedures have been described, with posterior tibial tendon (PTT) transfer remaining a reliable and biomechanically effective option for restoring active dorsiflexion [2,3]. The procedure was first described by Ober in 1933 and later modified by Putti in 1937 to pass the tendon through the interosseous membrane, a technique further popularized by Watkins in 1954 [2,4,5].

Successful PTT transfer requires careful consideration of the route of transfer, insertion site, fixation method, and whether to pass the tendon superficial or deep to the extensor retinaculum. Thorough preoperative assessment is essential to determine the optimal strategy for maximizing postoperative ankle dorsiflexion and functional recovery.

We present a case of PTT transfer combined with forefoot reconstruction procedures for foot drop and forefoot pain secondary to CMT disease. This case demonstrates successful restoration of functional dorsiflexion and patient satisfaction.

## Case presentation

Patient history and initial presentation

The patient is a 59-year-old female with a history of CMT1A, diabetes mellitus, and peripheral neuropathy. She presented to our clinic in May 2023 with complaints of bilateral foot pain, with increased pain in the left foot. The patient reported that her toes were curling and rubbing against her shoes, causing difficulty walking. She had previously been followed in the clinic for left posterior heel pain.

**Figure 1:**
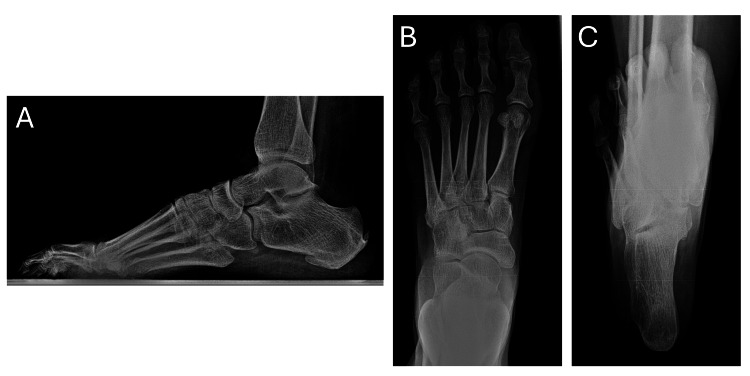
Initial consultation radiographs Pre-procedure left foot radiographs, including lateral, AP, and calcaneal axial views, taken in May 2014. (A) Lateral view reveals flexion contractures of digits 2-5. (B) AP view demonstrates decreased first metatarsal protrusion distance. (C) Calcaneal axial view shows an 8° calcaneal varus deformity.

Prior weight-bearing radiographs performed in May 2014 (Figure 1) demonstrated a posterior calcaneal spur at the insertion of the Achilles tendon on the calcaneus, an 8° calcaneal varus deformity, flexion contractures of digits 2-5, and decreased first metatarsal protrusion distance. In July 2014, she underwent surgical intervention consisting of a gastrocnemius recession and retrocalcaneal exostectomy with detachment and reattachment of the Achilles tendon with the senior author, a podiatric foot and ankle surgeon. Final postoperative weight-bearing radiographs obtained in October 2014 revealed two anchors in the posterior calcaneus. Her recovery after the operation was unremarkable.

Reevaluation and preoperative assessment

The patient returned to the clinic in October 2022 with a new chief complaint of left lateral foot pain and difficulty walking, which began in May 2022. She denied any history of trauma to the common peroneal nerve or falls. Physical examination revealed diminished Achilles and patellar reflexes. Musculoskeletal assessment showed dorsiflexion and eversion strength of 3/5, while plantarflexion and inversion strength were 5/5. Pain was noted on palpation of the fourth and fifth tarsometatarsal joints. The subtalar and remaining tarsal joints were supple, with an adequate range of motion and no pain.

**Figure 2:**
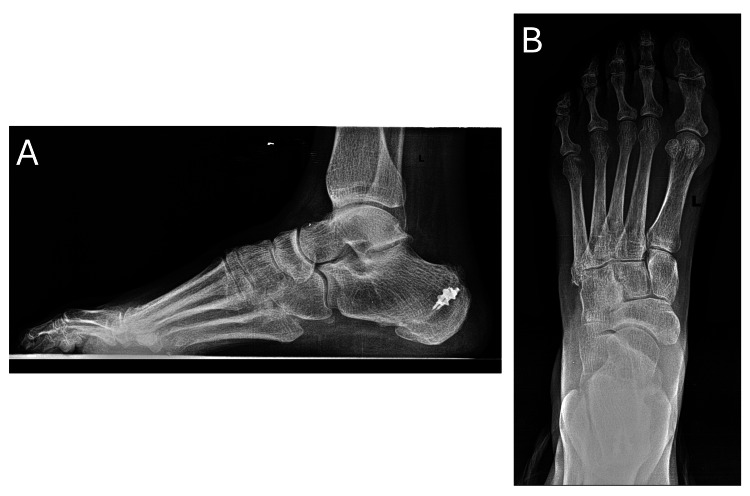
Radiographs prior to PTT transfer Pre-procedure left foot radiographs (lateral and AP) performed in October 2022. (A) Lateral view shows two anchors from prior retrocalcaneal exostectomy with detachment and reattachment of the Achilles tendon. (B) AP view remains unchanged from the initial encounter. PTT, posterior tibial tendon

Weight-bearing radiographs obtained at this visit (Figure 2) revealed two anchors from the previous Achilles tendon repair. Asymmetric narrowing of the fourth and fifth tarsometatarsal joints was noted. Hammertoe deformities were present in digits 2-5, along with decreased first metatarsal protrusion distance. Conservative treatment with a custom AFO was recommended.

At follow-up three weeks later, the patient reported facial numbness and increased weakness in the extremity. She was subsequently referred to a neurologist for evaluation of possible neuromuscular disease.

Diagnosis and preoperative status

After a thorough interdisciplinary evaluation, the patient was diagnosed with CMT1A in May 2023. She returned to the clinic in August 2024 with a chief complaint of left foot pain and limited range of motion. Physical examination revealed semirigid hammertoe contractures due to extensor substitution, with muscle strength similar to her October 2022 visit. The patient continued to experience pain with the use of her custom AFO, which limited her activities of daily living.

Surgical intervention

The patient elected to proceed with surgical intervention and underwent a PTT transfer through the interosseous membrane of the left foot, percutaneous tendoachilles lengthening, arthrodesis with pinning of digits 2-4, arthroplasty of the fifth digit, and Weil osteotomies of the second and third metatarsals of the left foot in September 2024.

Surgical procedure

Zone 3 tendoachilles lengthening was performed to address gastrocnemius-soleus equinus deformity, resulting in an increased dorsiflexion range of motion to approximately 8° above neutral. A 5-cm longitudinal incision was made from the medial malleolus to the navicular tuberosity along the course of the PTT. The PTT was released from its insertion on the navicular tuberosity, and a separate incision was made over the posteromedial aspect of the leg, approximately 10 cm proximal to the medial malleolus. The PTT was then transferred into the proximal posteromedial incision.

A third incision was made on the anterolateral aspect of the tibia, approximately 5 cm above the anterior aspect of the ankle joint. A large right-angle forceps was passed through this anterolateral incision in a posteromedial direction until the interosseous membrane was reached. An opening was created in the interosseous membrane, and the PTT was passed through the membrane and out of the anterolateral incision. A small linear incision was made in the tibialis anterior tendon, and the PTT was pulled through in a posterior-to-anterior fashion.

A fourth incision was made dorsally over the intermediate cuneiform. The PTT was transferred subcutaneously from the anterolateral leg incision into the dorsal midfoot incision. The tendon was tunneled subcutaneously rather than deep to the retinaculum to maximize dorsiflexion potential. The foot was held in an everted and maximally dorsiflexed position, approximately 8° above neutral, while the PTT was tensioned by removing slack and secured with a 6 × 23 mm interference screw into the intermediate cuneiform. This provided physiologic tension to maximize tendon function. The suture ends of the tendon were anchored to the plantar aspect of the foot using a sterile button.

Correction of second- and third-plantar metatarsal prominence, metatarsophalangeal joint subluxation, and hammertoe deformity was achieved via Weil osteotomy of the second and third metatarsals, along with arthrodesis of the proximal interphalangeal joints of digits 2-4 with pinning, and arthroplasty of the fifth digit.

Postoperative management

Postoperative care included two weeks of non-weight-bearing in a posterior splint, followed by five weeks of non-weight-bearing in a cast. On postoperative day 49, the surgical pins were removed, and manual muscle testing demonstrated dorsiflexion strength of 3/5. The patient then transitioned to progressive weight-bearing in a controlled ankle motion boot for four weeks and began physical therapy.

Physical therapy included joint mobilization, manual resisted range of motion, neuromuscular re-education, stretching, strengthening, and conditioning exercises. On postoperative day 68, the patient was instructed to transition to supportive shoe gear and gradually increase activities over four to six weeks.

In December 2024, postoperative day 106, the patient reported satisfaction with the results and noted she was able to tap her foot, a movement she had never been able to perform previously. The patient returned to the clinic in October 2025, 13 months postoperatively, wearing regular shoes. She reported being pleased with the results and able to resume full functional activity.

**Figure 3:**
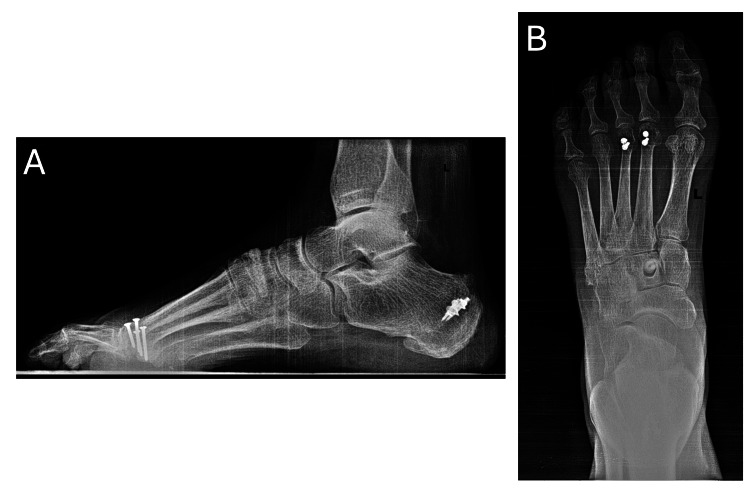
Final postoperative radiographs of the left foot Postoperative AP and lateral radiographs, October 2025. (A) Lateral view showing the interference screw in the intermediate cuneiform, hardware in the second and third metatarsal heads, and bone anchors in the posterior calcaneus. (B) AP weight-bearing radiograph demonstrating a normalized metatarsal parabola and the interference screw within the intermediate cuneiform.

**Figure 4:**
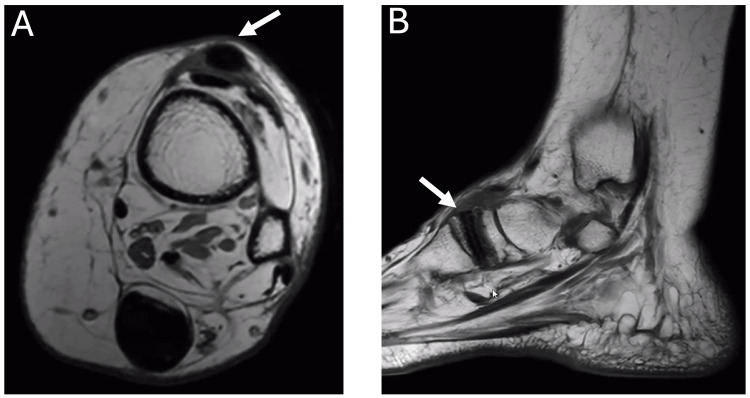
MRI of left ankle, October 2025 (A) Axial view showing the subcutaneous transfer of the PTT on the anterior ankle. (B) Sagittal view demonstrating integration of the tendon-bone interface postoperatively. White arrows indicate the PTT transfer in the left foot. PTT, posterior tibial tendon

Weight-bearing radiographs (Figure 3) demonstrated well-maintained hardware, a normal metatarsal parabola, and two-screw fixation in the second and third metatarsals. The interference screw from the PTT transfer was visualized within the intermediate cuneiform, along with two calcaneal bone anchors from the prior retrocalcaneal Achilles tendon surgery. Postoperative MRI sections (Figure 4) illustrate the PTT transfer in the anterior ankle with integration of the bone-tendon interface in the intermediate cuneiform.

**Figure 5:**
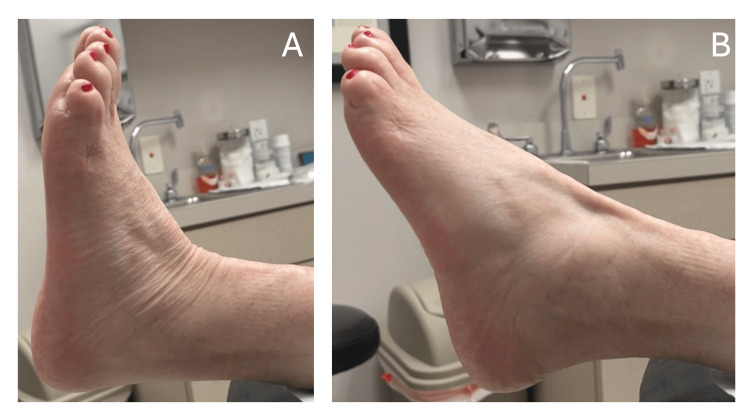
Clinical evaluation, October 2025 Thirteen-month postoperative clinical evaluation. (A) Active dorsiflexion, approximately 4° above neutral. (B) Foot in a relaxed resting position.

The patient has returned to full functional activity, including working out. Manual muscle testing of the left extremity revealed dorsiflexion strength of 4/5 and plantarflexion strength of 5/5. Well-maintained range of motion of the left ankle was observed, with active dorsiflexion demonstrated in Figure 5. The patient is to return to the clinic as needed.

## Discussion

Foot drop is a debilitating gait abnormality that significantly impairs functional mobility and quality of life. It can arise from a wide spectrum of underlying pathologies, including neuromuscular disorders, peripheral nerve injuries, traumatic disruption of the anterior musculature, and anterior compartment syndrome. The resulting inability to actively dorsiflex the ankle not only compromises gait efficiency but also increases the risk of falls and secondary musculoskeletal complications. Among hereditary neuropathies, CMT disease is one of the most common etiologies and is frequently associated with progressive weakness of the anterior compartment, calcaneal varus deformity, and eventual foot drop.

PTT transfer has long been regarded as a reliable and biomechanically sound method for restoring active ankle dorsiflexion in patients with CMT-related foot drop. The procedure was popularized by Watkins by taking advantage of the PTT’s robust strength, reliable excursion, and favorable line of pull when redirected to the dorsum of the foot [2,4]. The fundamental surgical principle involves rerouting a functioning tendon with sufficient contractile potential to compensate for the compromised anterior compartment musculature. Optimal outcomes are associated with a strong PTT, flexible rearfoot and ankle joints, and adherence to comprehensive postoperative rehabilitation.

While alternative tendon transfers, such as the peroneus longus or flexor digitorum longus, may be considered in selected clinical scenarios, PTT transfer remains the most widely utilized option for achieving consistent improvements in dorsiflexion and gait. Numerous studies in podiatric and orthopedic literature have demonstrated satisfactory restoration of ankle dorsiflexion strength, improved swing-phase clearance, and measurable gait normalization following PTT transfer. For example, Chung et al. evaluated 14 patients with foot drop treated with PTT transfer [6]. Ten patients were able to ambulate postoperatively without the use of an AFO. Our case highlights the benefits of PTT transfer in the CMT population, where progressive neuromuscular deterioration is expected, as well as its potential to allow patients to transition out of an AFO for improved quality of life.

A 2021 review by Wakefield et al. evaluated several variations of PTT transfer. These procedure variations included transfer of the posterior tibial tendon through the interosseous membrane, circumtibial routing of the posterior tibial tendon, and the Bridle procedure [7]. Their findings highlighted important distinctions in surgical technique, postoperative biomechanics, and complication profiles. For instance, the circumtibial route, while technically simpler, was associated with a higher incidence of anterior ankle bowstringing when the tendon was positioned subcutaneously. Nevertheless, patients in the reviewed series experienced minimal discomfort, and functional outcomes remained satisfactory. In our case, the patient demonstrated similar postoperative tolerance, comfort in standard shoe gear, and significant improvement in dorsiflexion strength. Wakefield et al. also emphasized the importance of tailoring the insertion point based on the degree of preexisting varus deformity, noting that more lateral insertion points may optimize mechanical advantage in patients with pronounced calcaneal varus alignment [7].

Further insight into tendon insertion techniques was provided by Faisal et al. in 2024, who compared outcomes between the classic Barr’s technique and a modified Barr’s technique [8]. Their study demonstrated a higher rate of dorsiflexion normalization with the modified technique, paralleling the functional improvements observed in our patient. Interpretation of these results should, however, be tempered by the study’s relatively short follow-up period of six months and its limited patient cohort.

Transfer of the PTT superficial to the extensor retinaculum was first described by Root et al., who reported successful functional outcomes but did not address the potential complication of bowstringing [9]. A cadaveric study by D’Astous et al. demonstrated that superficial transfers increase the tendon’s moment arm by positioning it farther from the ankle’s center of rotation, theoretically enhancing resistance to dorsiflexion loads [10]. Our case provides clinical correlation to these findings, as significant dorsiflexion strength was observed 13 months postoperatively. Conversely, studies such as Wagner et al., comparing circumtibial, above-retinaculum transmembranous, and under-retinaculum routes, found comparable gliding resistance in superficial versus deep transfers, suggesting that technique selection may require further in vivo validation [11].

A more comprehensive examination of the literature was presented in a systematic review by Stevoska et al. [12], which assessed 37 studies involving PTT transfers for foot drop. Tendon-to-tendon fixation was the most frequently utilized method in earlier reports; however, more recent studies document increasing use of tendon-to-bone fixation with outcomes comparable to or better than traditional approaches. In our patient, tendon-to-bone fixation was performed using an interference screw placed in the intermediate cuneiform. Appropriate tensioning of the tendon transfer was achieved according to the surgical technique described by Mayer [5]. Postoperative MRI confirmed successful tendon incorporation at the bone interface, supporting the viability of this method. Stevoska et al. further reported that 91% of patients were ultimately able to ambulate without an AFO, illustrating the procedure’s capacity to restore meaningful functional independence [12].

Quality-of-life outcomes also play a central role in surgical decision-making. A 2011 study by Steinau et al. reported that, despite postoperative complications, 80.6% of patients indicated they would undergo the procedure again [13]. Rehabilitation remains a cornerstone of postoperative success, with gradual, structured strengthening and gait retraining critical to ensuring proper tendon recruitment and optimizing the new line of pull. At 13 months postoperatively, the patient demonstrated functional recovery of dorsiflexion, returned to normal activities of daily living without an AFO, and achieved outcomes consistent with favorable results reported in the literature.

## Conclusions

This case reinforces the effectiveness of PTT transfer combined with forefoot reconstruction as a reliable surgical option for foot drop secondary to CMT disease. It also highlights the importance of careful surgical technique selection, appropriate fixation strategy, and structured postoperative rehabilitation in achieving durable functional recovery. The procedure restores active dorsiflexion, improves gait mechanics, and enhances the patient’s quality of life. As surgical methods continue to evolve, the growing body of evidence supports tendon-to-bone fixation and individualized routing strategies as promising refinements that may further optimize patient outcomes.
